# Adenosine-Mediated Enteric Neuromuscular Function Is Affected during Herpes Simplex Virus Type 1 Infection of Rat Enteric Nervous System

**DOI:** 10.1371/journal.pone.0072648

**Published:** 2013-08-27

**Authors:** Chiara Zoppellaro, Anna Bin, Paola Brun, Serena Banzato, Veronica Macchi, Ignazio Castagliuolo, Maria Cecilia Giron

**Affiliations:** 1 Department of Pharmaceutical and Pharmacological Sciences, University of Padova, Padova, Italy; 2 Department of Molecular Medicine, University of Padova, Padova, Italy; Cincinnati Childrens Hospital Medical Center, United States of America

## Abstract

Adenosine plays an important role in regulating intestinal motility and inflammatory processes. Previous studies in rodent models have demonstrated that adenosine metabolism and signalling are altered during chronic intestinal inflammatory diseases. However, the involvement of the adenosinergic system in the pathophysiology of gut dysmotility associated to a primary neurodysfunction is still unclear. Recently, we showed that the neurotropic *Herpes simplex* virus type-1 (HSV-1), orally inoculated to rodents, infects the rat enteric nervous system (ENS) and affects gut motor function without signs of systemic infection. In this study we examined whether changes in purinergic metabolism and signaling occur during permanent HSV-1 infection of rat ENS. Using isolated organ bath assays, we found that contraction mediated by adenosine engagement of A_1_ or A_2A_ receptors was impaired at 1 and 6 weeks post-viral administration. Immunofluorescence studies revealed that viral infection of ENS led to a marked redistribution of adenosine receptors: A_1_ and A_2B_ receptors were confined to the muscle layers whereas A_2A_ and A_3_ receptors were expressed mainly in the myenteric plexus. Viral-induced ENS neurodysfunction influenced adenosine metabolism by increasing adenosine deaminase and CD73 levels in longitudinal muscle-myenteric plexus with no sign of frank inflammation. This study provides the first evidence for involvement of the adenosinergic system during HSV-1 infection of the ENS. As such, this may represent a valid therapeutic target for modulating gut contractility associated to a primary neurodysfunction.

## Introduction

Gastrointestinal neuromuscular diseases (GINMD), including chronic idiopathic intestinal pseudo-obstruction and irritable bowel syndrome, are a clinically heterogeneous group of diseases presumed to result from morpho-functional alterations of the enteric nervous system (ENS), in the absence of evident structural or biochemical abnormalities. These disorders are characterized by motor impairments and abnormal visceral perception and secretion, with high morbidity and occasional fatal outcome [1], [2]. Infectious agents, such as neurotropic viruses, have been suggested as etiologic factors involved in ENS disruption [3]. However, there is only scarce evidence proving a direct causative role of these viruses in functional bowel disorders; these are based on reports of disease onset in patients after a viral infection, or on detection of viral genomes in the ENS of seriously ill patients [4], [5].

Among several neurotropic viruses *Herpes simplex* virus type 1 (HSV-1) is a pathogen which merits consideration as a potential etiologic factor of GINMD. Indeed, HSV-1 has the ability to remain latent in host neurons for life. Reactivation from the latent state results in productive HSV-1 infection that ultimately leads to the lytic destruction of distal epithelial cells. During latency HSV-1 resides in neurons by producing only non-coding viral transcripts, collectively known as latency associated transcripts (LATs). HSV-1 replication involves the expression of viral genes in a tightly regulated, ordered cascade which begins with production of immediate-early genes α (e.g. infected cell proteins, ICP0, ICP4, ICP22, and ICP27) responsible for regulating viral gene expression during subsequent phases of the replication cycle [6]. Gesser et al. (1994, 1995, 1996) showed that in mice HSV-1 orally inoculated was able to establish latency in the nodose ganglia of the vagus nerve or spread through the myenteric, submucosal, and periglandular plexuses leading to progressive inflammatory disease and death [7]–[9]. HSV-1 shedding from the oropharyngeal mucosa occurs in chronically infected human subjects thus facilitating virus passage to the gastrointestinal tract mucosa where it infects the nodose and celiac ganglia.

Our research group has established a novel animal model of persistent HSV-1 infection in the ENS, using a protocol characterized by an intranasal low viral inoculum to create a latent HSV-1 infection in the central nervous system, followed after four weeks by an intragastric viral administration [11]. In this model latent HSV-1 persists in the ENS for several weeks after oral challenge, which leads to gut motor abnormalities in the absence of frank inflammation. So far, the mechanism(s) through which primary defects in ENS are responsible for bowel dysfunction, including motor abnormalities, remains poorly understood. Our animal model displays several interesting features which can be capitalized upon to address such basic biological questions.

Adenosine plays a prominent role in intestinal functions, modulating the complex interplay between ENS, smooth muscle and epithelial barrier function in physiological and pathological conditions [12], [13]. Adenosine exerts its biological actions through G-protein-coupled receptor subtypes designated as A_1_, A_2A_, A_2B_ and A_3_, each with distinct affinities for adenosine and distribution and function in the gut. Under normal conditions, adenosine concentration is dependent on processes such as intracellular and extracellular biosynthesis, cellular release, re-uptake and metabolism [14]. However, under adverse conditions, such as hypoxia or inflammation, production of extracellular adenosine rises dramatically and the nucleoside appears to have a pivotal role in neuromuscular function and in the prevention of inflammatory responses [12], [13]. Besides its recognized role in regulating inflammatory responses, adenosine appears to be involved in viral infection. Inhibition of the A_1_ receptor (A_1_R) pathway suppresses HSV-1 replication, whereas increased activity of ecto-5′nucleotidase (CD73) may be a potential evasion strategy developed by the *Herpesviridae* cytomegalovirus [15], [16]. Little is known, however, about possible involvement of the adenosinergic system in the pathophysiology of gut dysmotility associated with a primary neurodysfunction. The animal model we have developed is unique when placed alongside the large body of existing data on gastrointestinal neurodysfunction, in which ENS damage arises from chemically- or parasite infection-induced mucosal inflammation and not from primarily affected myenteric ganglia [17], [18]. The present study was therefore designed to investigate whether in rat ileum adenosine metabolism and signaling are affected during HSV-1 infection of ENS, in terms of contractile responses to adenosine and distribution of its receptor subtypes and metabolic enzymes, namely adenosine deaminase (ADA) and CD73.

## Methods

### Reagents

Purified rabbit anti-human ADA polyclonal antibody was purchased from SantaCruz Biotechnology Inc. (catalogue n. sc-25747, Heidelberg, Germany), rabbit anti-rat A_1_R, rabbit anti-canine A_2A_R, rabbit anti-human A_2B_R, and rabbit anti-rat A_3_R polyclonal antibodies were from AlphaDiagnostic International (San Antonio, USA), and mouse anti-rat CD73 was from BD Biosciences (Milan, Italy). Alexa Fluor 488 goat anti-rabbit immunoglobulin G and Alexa Fluor 488 goat anti-mouse immunoglobulin G were obtained from Invitrogen (Milan, Italy). Adenosine, N^6^-cyclopentyladenosine (CPA), 2-*p*-(2-carboxyethyl) phenethylamino-5′-*N*-ethyl carboxamidoadenosine hydrochloride hydrate (CGS-21680), 1,3-dipropyl-8-cyclopentylxanthine (DPCPX) and 4-(2-[7-amino-2-(2-furyl) [1], [2], [4]triazolo[2,3-a] [1], [3], [5]triazin-5-ylamino]ethyl) phenol (ZM 241385) were dissolved in dimethyl sulfoxide (DMSO) and further diluted in Tyrode’s solution. All the other drugs were dissolved in distilled water. DMSO concentration in organ bath never exceeded 0.5% with no effect on contractile responses of ileum preparations. Working solutions were freshly prepared by diluting stock solutions in Krebs on the day of the experiment. CPA, CGS-21680, DPCPX and ZM 241385 were purchased from Tocris Bioscience (Bristol, UK). Unless otherwise specified, all chemicals used were purchased from Sigma Aldrich (Milan, Italy).

### Animals, viral Inoculation and Infection

HSV-1 strain 16 propagation was carried out as previously described on Vero cells [11]. HSV-1 inoculation in male Wistar rats (350–450 g body weight; Harlan, Italy) was performed by a two-step infection protocol, consisting of an initial intranasal (IN) instillation followed four weeks later by an intragastric inoculum. Animals were first anesthetized with tiletamine–zolazepam (20 mg/kg intramuscular; Zoletil, Virbac, Italy) and xylazine (5 mg/kg intramuscular; Bayer S.p.A., Italy). HSV-1 intranasal administration (10^3^ plaque forming units, pfu in 20 µl phosphate buffered saline, PBS) was performed using a micropipette to drop virus solution onto rat external nares. After four weeks, intranasally infected rats were administered 10^8^ pfu HSV-1 via intragastric gavage using a 24-gauge, 9-cm catheter. Before and after treatments all animals had free access to standard laboratory chow and tap water and were housed, three in a cage, in temperature-controlled rooms on a 12-h light cycle at 22–24°C and 50–60% humidity, and were observed daily for signs of disease. At either 1 or 6 weeks post-intragastric inoculation, rats were killed by decapitation. Control animals were injected with equal volumes of Vero cell lysate (sham infection) and killed at matching time points. No histological and functional changes were observed between time-matched sham animals which were thus considered as a single control group, designated as sham group. All experimental protocols were approved by the Animal Care and Use Committee of the University of Padova, and were in compliance with the national and European guidelines for handling and use of experimental animals.

### Histology, Inflammatory Cytokine Analysis and Myeloperoxidase Activity

Specimens of distal ileum (5–10 cm from ileocecal valve) were fixed in 10% buffered formalin for 24 h, and then embedded in paraffin. Sections (5-µm-thick) were stained with haematoxylin & eosin. Slides (n = 3 rat for each experimental group) were assessed by a observer blinded to the treatment groups. Ileal segments were homogenized in ice-cold 50 mM phosphate buffer, pH 6.0, containing 0.5% hexadecyltrimethylammonium bromide to prevent the pseudoperoxidase activity of haemoglobin and to solubilise membrane-bound myeloperoxidase (MPO). Homogenates were centrifuged 10,000 g (10 min at 4°C) and MPO activity was measured by colorimetric assay [11]. All samples were examined within 2 days of collection. Segments of small intestine were collected from sham or infected animals and the longitudinal muscle myenteric plexus (LMMP) homogenized in PBS (1∶10 wt/vol) supplemented with protease inhibitors (1 mmol/L phenylmethylsulfonyl fluoride, 10 µg/mL aprotinin, 10 µg/mL leupeptin). After centrifugation (10,000 × *g* for 10 min at 4°C), interleukin-2 (IL-2), interferon-γ (IFN-γ), and tumor necrosis factor-α (TNF-α) were quantified by enzyme-linked immunosorbent assay (Biosource, Milan, Italy). Data were normalized to total protein content using the bicinchoninic acid method [19].

### Myenteric Ganglia Isolation

Myenteric ganglia were isolated as previously described [11]. Briefly, LMMPs from sham or infected animals were stripped and digested with protease (1 mg/mL) and collagenase type IV (1.4 mg/mL) at 37°C for 60 min. The resulting cell suspensions were washed and myenteric ganglia were collected under a dissecting microscope and snap-frozen in liquid nitrogen for subsequent DNA and RNA extraction.

### DNA Extraction and Polymerase Chain Reaction for HSV-1 TK

Isolated myenteric ganglia were homogenized in 1∶10 wt/vol DNA lysis buffer (1.25% wt/vol sucrose, 0.3% vol/vol NP-40, 10 mmol/L NaCl, 3 mmol/L MgCl_2_, 20 mmol/L Tris-HCl, pH 7.4) and then incubated at 56°C in 10% (wt/vol) sodium dodecyl sulfate and proteinase K (200 µg/mL). DNA was purified by phenol/chloroform extraction and precipitated with ethanol. To assess the presence of the HSV-1 genome, quantitative polymerase chain reaction (PCR) was performed using an ABI Prism 7700 Sequence Detection System (PerkinElmer, Monza, Italy) on 200 ng DNA using TaqMan Universal PCR Master Mix (Applied Biosystems, Monza, Italy) and primers for HSV-1 thymidine kinase (TK). Rat β-globin was used as extraction and loading control. Oligonucleotide primer sequences and amplification conditions are reported in Table 1.

**Table 1:** PCR primers and probe sequences, PCR annealing temperature, and size of the amplicons.

Gene	Direction	Sequence	Temperature (°C)	Amplicon size (bp)
TK	forward	TAGCCCGGCCGTGTGACA		
	reverse	CATACCGGAACGCACCACACAA	60	302
	probe	ATCCACGCAACTGAGCAC		
β-globin	forward	AGGAAGCCACTCTAGGGAGC		
	reverse	AGAACAGAGTGAGCGGGAGA	60	100
	probe	AAGCAGGCGGCCGAGGGC		
GADPH	forward	AAGTATGATGACATCAAGAAGG		
	reverse	ACTAAAGGGCATCCTGGGCT	60	166
LAT	forward	GACAGCAAAAATCCCCTGAG		
	reverse	ACGAGGGAAAACAATAAGGG	56	175
ICP4	forward	ATGACGGGGACGAGTACGAC		
	reverse	ACGACGAGGACGAAGAGGAT	60	223

TK, thymidine kinase; GAPDH, glyceraldehyde-3-phosphate dehydrogenase; LAT, latency associated transcript; ICP4, infected cell protein 4.

### RNA Extraction and Quantitative Real-time PCR for viral Genes

The SV total RNA isolation system (Promega) was used to extract total RNA from isolated myenteric ganglia according to the manufacturer’s protocol. Myenteric ganglia were homogenized in lysis buffer and contaminating DNA was removed by DNase I treatment (Promega). Total RNA (3 µg) was reverse transcribed to cDNA using random primers and MuLV reverse transcriptase (Applied Biosystems, Foster City, CA). To study the HSV-1 replication cycle, 5 µL of the reverse-transcription reactions were subjected to real-time quantitative polymerase chain reaction (qPCR) using primers for immediate early gene (infected cell protein, ICP4) and latency associated transcripts (LATs). qPCR was performed on an ABI PRISM 7700 Sequence Detection System (Perkin Elmer, Norwalk, CT) using SYBR Green (Applied Biosystems). The primer-dimers formation for each oligonucleotide set was ruled out by generating melt-curve profiles. For each sample, the amounts of the targets and endogenous reference (glyceraldehyde 3-phosphate dehydrogenase, GADPH) were extrapolated from a standard curve prepared using serially-diluted correspondent cDNAs subcloned into the pGEM-T vector (Promega). Oligonucleotide primer sequences and amplification conditions are reported in Table 1.

### Recording of Mechanical Activity

A 30-cm segment of distal ileum proximal to the ileocecal junction was removed and placed in Tyrode’s solution (in mM: NaCl 136, KCl 2.7, CaCl_2_·2H2O 1.4, MgCl_2_·6H_2_O 0.49, NaH_2_PO_4_ 0.32, NaHCO_3_ 12, glucose 5). Ileal segments (1.5 cm) were mounted vertically in 10-mL organ baths containing Tyrode’s solution, maintained at 37°C and aerated with 95% O_2_ and 5% CO_2_. In vitro contractility studies were performed as previously described [14]. Briefly, changes in muscle tension were recorded by isometric force transducers (World Precision Instruments, 2Biological Instruments, Varese, Italy) connected to a quad bridge amplifier and PowerLab 4/30 data acquisition system using LabChart6 software (ADInstruments, Oxford, UK). After 45 min-equilibration smooth muscle segments were stretched passively to an initial tension of 1 g and brought to their optimal point of length-tension relationship using 1 µM carbachol.

### Experimental Protocols

In basal conditions concentration-response curves (100 µM - 1.25 mM) for adenosine were constructed by cumulative addition of the nucleoside. Responses were expressed as gram tension/gram dry tissue weight of ileal segments. Adenosine-evoked concentration-response curves (plotted against log of adenosine concentration) were subjected to a non-linear regression analysis (fitted to a sigmoidal equation) to calculate EC_50_ and Emax values. Contractile effects of adenosine (1 mM) were investigated in the presence or absence of atropine (1 µM) or tetrodotoxin (1 µM, TTX; Tocris Bioscience, Bristol, UK) or DPCPX (0.01 µM), an A_1_ purinoceptor antagonist, or ZM 241385 (0.01 µM), an A_2A_ purinoceptor antagonist, which remained in contact with the tissue for 20 min before repeating the adenosine curve. The effects of the A_1_ purinoceptor agonist CPA (10 µM) and the A_2A_ purinoceptor agonist CGS-21680 (10 µM) were evaluated in the absence or presence of atropine or TTX, respectively.

### Immunofluorescence Imaging

Distal ileum segments were frozen in optimal cutting temperature (OCT) mounting medium. From each ileal specimen of each experimental animal (n = 3 rats for each group) 100 sequential 5 µm-cross-sections were cut on a cryostat and 1/20 sections for each ileal specimen were subjected to immunohistochemistry as previously described [8]. Briefly, sections from sham and infected animals were fixed for 15 min in 4% paraformaldehyde in PBS, washed in Tris-buffered saline, treated with normal goat serum, and incubated with anti-ADA (1∶100), anti-CD73 (1∶500), anti-A_1_R (1∶300), anti-A_2A_R (1∶300), anti-A_2B_R (1∶200) and anti-A_3_R (1∶400) for 1 h at room temperature. After Tris-buffered saline wash, sections were incubated for 1 h with Alexa Fluor 488 fluorescein isothiocyanate-labeled secondary antibody (1∶1000). Negative controls were performed by incubating sections with isotype-matched control antibodies at the same concentration as primary antibody and/or pre-incubating each antibody with 200-fold molar excess of the corresponding blocking peptide. For each section, five randomly selected microscopic fields were acquired through x63 oil immersion objective (Plan Neofluar) of a Leica TCSNT/SP2 confocal microscope (Leica Microsystem, Milan, Italy).

### Western Blot

Whole thickness ileum without mucosa was homogenized in nondenaturing RIPA buffer (150 mM NaCl, 50 mM Tris-HCl, 0.25% wt/vol sodium deoxycholate, 0.1% Nonidet P-40, 100 µM NaVO_4_, 1 mM NaF, 1mM phenylmethylsulfonyl fluoride, 10 µg/ml aprotinin, 10 µg/ml leupeptin). Particulate material was removed by centrifugation. Protein concentration was determined in each sample by the Bradford method with a commercially available kit (Protein Assay Kit; Bio-Rad Laboratories, Hercules, CA). Aliquots of 30 µg-protein were separated through a SDS-polyacrylamide gel electrophoresis and then transferred onto a nitrocellulose membrane, as described previously [8]. Membranes were probed with polyclonal anti-ADA antibody (1∶200) and then incubated with the proper horseradish peroxidase-conjugated secondary antibody. Bands were visualized using enhanced chemiluminescence (Millipore, Milan, Italy). Images were captured using a Hyper Film MP (GE Healthcare, Milan, Italy) and densitometry was performed using NIH Image J software. To ensure equal loading and accuracy of changes in protein abundance, protein levels were normalized to β-actin.

### Data Analysis

All results were expressed as mean ± SEM. Ileal preparations included in each experimental group were obtained from separate animals, and hence the number of experiments refers to the number of animals assigned to each experimental group. Unless stated, for each animal a number of four ileal specimens were used for each experimental test. Adenosine responses were fitted to sigmoid curves (GraphPad Prism 3.03, San Diego, CA), and EC_50_ values with 95% confidence limits (CLs) were determined. Statistical analysis was performed by paired or unpaired Student’s t-tests or one-way ANOVA followed by Neuman-Keuls multicomparison test when appropriate, using GraphPad Prism 3.03. *P*-values of <0.05 were considered statistically significant.

## Results

### Assessment of HSV-1 Infection of ENS

**Figure 1:**
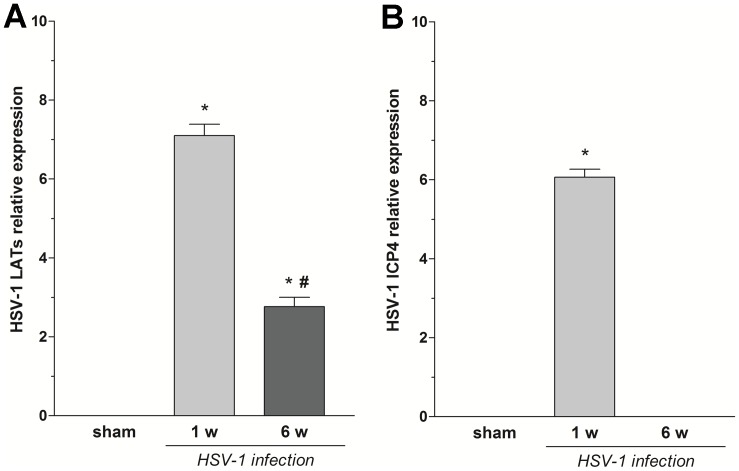
Nature of HSV-1 infection in the ENS. RNA was purified from myenteric ganglia of sham and HSV-1 infected rats at 1 and 6 weeks (w) after HSV-1 intragastric administration and the relative expression of HSV-1 LATs (A), and of the very early gene ICP4 (B) was determined by qPCR. Glyceraldehyde-3-phosphate dehydrogenase was used as internal control. Data are expressed as means ± SEM (two tissue samples from n = 3 animals for each experimental group). *P<0.01 compared to sham rats; ^#^P<0.01 compared to infected rats at 1 week post-intragastric administration.

**Figure 2:**
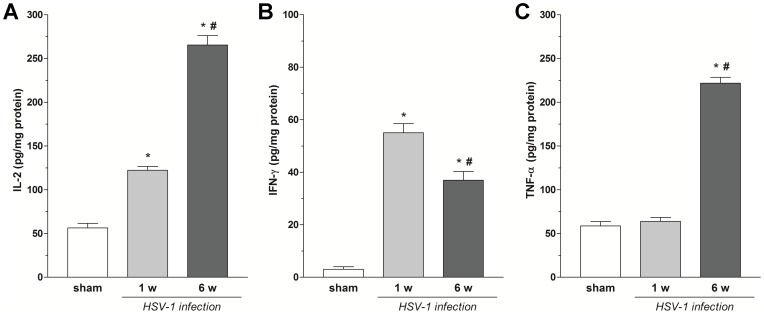
Inflammatory markers in LMMP during HSV-1 infection. IL-2 (A), TNF-α (B), IFN-γ (C) levels were determined in the LMMPs of sham and HSV-1 infected rats at 1 and 6 weeks (w) after HSV-1 intragastric inoculum (two tissue samples from n = 3 animals for each experimental group). Data are expressed as means ± SEM pg/mg protein.*P<0.01 compared to sham rats; ^#^P<0.01 compared to infected rats at 1 week post-intragastric administration.

Six weeks post HSV-1 intragastric inoculum HSV-1 TK DNA was still detectable in myenteric ganglia, although viral DNA copy number was reduced as compared to the first week after intragastric administration (data not shown). To define the nature of HSV-1 infection in the ENS, qPCR analysis of HSV-1 mRNA transcripts in the myenteric ganglia of sham and infected rats was performed. Expression of viral LATs was detected in isolated myenteric ganglia at 1 and 6 weeks after intragastric inoculation, whereas mRNA transcripts of the viral immediate early ICP4 gene were present only at 1 week post-viral inoculum (Figure 1). During the viral infection time-course no signs of illness were detected in infected rats. A preserved ileal architecture, together with no changes in ileal MPO activity confirmed the absence of inflammatory damage in HSV-1 infected animals (data not shown). However, there was a significant increase in IL-2 and IFN-γ levels in the intestinal LMMPs at 1 and 6 weeks after HSV-1 intragastric administration as compared to sham (Figure 2A and B), whereas TNF-α was increased significantly only at 6 weeks (Figure 2C).

### Effects of HSV-1 Infection on Adenosine-mediated Contraction

Isolated segments of the rat ileum displayed spontaneous activity consisting of phasic contractions with amplitude of 0.61±0.01 g and frequency of 25.8±0.25 cycles⋅min^−1^. The amplitude of spontaneous phasic contractions was significantly higher at 1 and 6 weeks (+43% and +26%, respectively) post-intragastric HSV-1 inoculum compared to sham.

**Figure 3:**
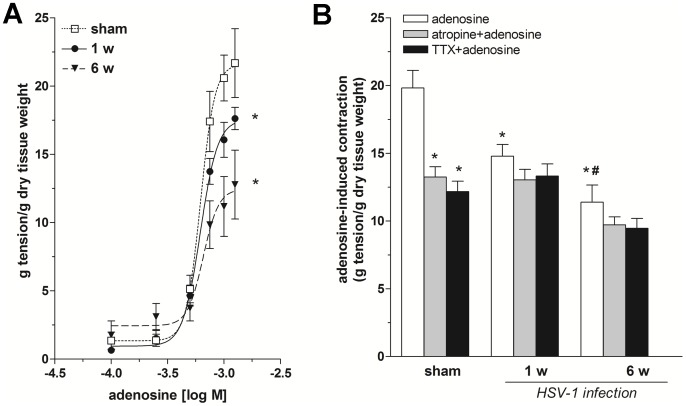
Effects of exogenous adenosine on ileal contractility during HSV-1 infection. (A) Concentration–response curves to adenosine in rat ileum from sham and infected rats at 1 and 6 weeks (w) after HSV-1 intragastric inoculum. Data are means ± SEM and are expressed as gram tension/gram dry tissue weight (four tissue samples from n = 6 animals for each experimental group). (B) Excitatory effects induced by 1 mM adenosine before and after treatment with atropine, a muscarinic receptor blocker (1 µM) or tetrodotoxin, a Na^+^ voltage-gated neural channel blocker (TTX, 1 µM). Data are means ± SEM and are expressed as g tension/g dry tissue weight (four tissue samples from n = 6 animals for each experimental group). *P<0.05 compared to the adenosine response in sham rats. ^#^P<0.05 compared to the adenosine response in infected rats at 1 week post-intragastric inoculum.

In order to avoid interference by HSV-1-induced anomalous cholinergic transmission [11] in adenosine-evoked motor responses, the adenosine response was directly evaluated on spontaneous basal contractility of ileal preparations. In physiological conditions, adenosine (100 µM–1.25 mM) produced a contraction that persisted throughout the contact time, reaching a maximum of 21.6 ± 1.4 g tension/g dry tissue weight at 1.25 mM, with an EC_50_ of 0.62 mM (95% CL 0.54–0.71 mM; Figure 3A). A significant downward shift of the concentration-response curves to adenosine and a consequent significant decrease in Emax values occurred in HSV-1 infected rats 1 and 6 weeks (EC_50_ = 0.62 mM, 95% CL 0.55–0.69 mM and Emax = 17.6±0.9 g tension/g dry tissue weight; EC_50_ = 0.65 mM, 95% CL 0.47–0.91 mM and Emax = 12.5±1.8 g tension/g dry tissue weight, respectively) post HSV-1 intragastric administration compared with sham animals (Figure 3A). The direct responses to post-junctional receptor activation by exogenous adenosine were evaluated in the presence of atropine (a muscarinic receptor antagonist) or TTX (a potent neuronal voltage-gated Na+ channels). In sham rats, pre-treatment with atropine or TTX significantly reduced adenosine-mediated contraction (−33 and −38%, respectively), indicating that contraction induced by adenosine is partially dependent on acetylcholine release from enteric cholinergic nerves through preferential activation of prejunctional adenosine receptors in the rat myenteric plexus (Figure 3B). In contrast to sham ileum, pre-treatment with atropine or TTX failed to affect adenosine-induced contraction during HSV-1 infection (Figure 3B). These results support the view that during HSV-1 infection impairment of adenosine modulation can reflect defects of both cholinergic neurons and extrajunctional adenosine receptor activation.

**Figure 4:**
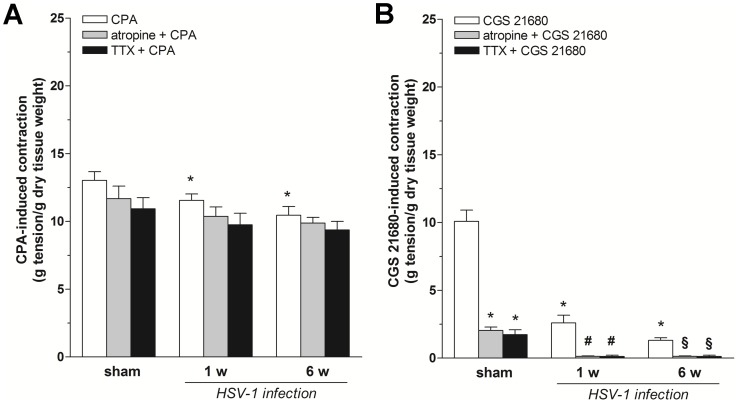
Effects of selective adenosine A_1_ or A2_A_ receptor agonist on ileal contractility during HSV-1 infection. Excitatory effects induced by 10 µM CPA (A) or 10 µM CGS-21680 (B) before and after treatment with atropine (1 µM) or tetrodotoxin (TTX, 1 µM) in rat ileum from sham and infected rats at 1 and 6 weeks (w) after HSV-1 intragastric administration. Data are means ± SEM and are expressed as g tension/g dry tissue weight (four tissue samples from n = 6 animals for each experimental group). *P<0.05 compared to the agonist response in sham rats. ^#^P<0.05 compared to the response of the respective agonist in infected rats at 1 week post-intragastric inoculum. ^§^P<0.05 compared to the respective agonist in infected rats at 6 weeks post-intragastric inoculum.

**Figure 5:**
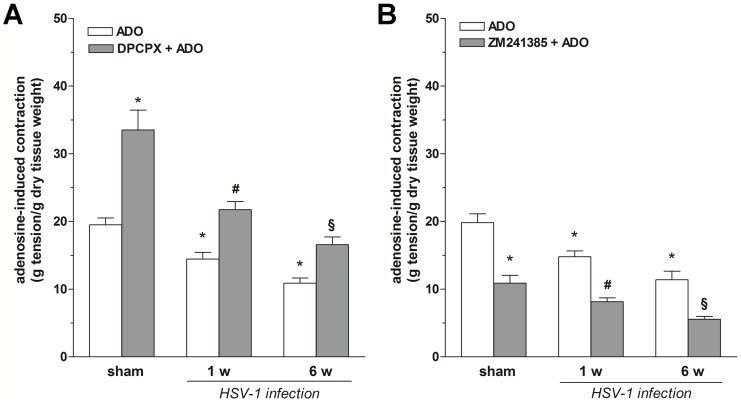
Effects of selective A_1_ or A_2A_ receptor antagonist on adenosine-induced ileal contraction during HSV-1 infection. Excitatory effects induced by adenosine at concentration of 1 mM before and after treatment with DPCPX (10 nM, panel A) or ZM241385 (10 nM, panel B) in rat ileum from sham and infected rats at 1 and 6 weeks (w) after HSV-1 intragastric administration. Data are means ± SEM and are expressed as g tension/g dry tissue weight (four tissue samples from n = 6 animals for each experimental group). *P<0.05 when compared to the response to adenosine in sham rats. ^#^P<0.05 when compared to the response to adenosine in infected rats at 1 week post intragastric inoculum. ^§^P<0.05 when compared to the response to adenosine in infected rats at 6 week post intragastric inoculum.

To characterize the P1 receptor subtypes involved in the observed response, the effects of CPA (A_1_R agonist) and CGS 21680 (A_2A_R agonist) on ileal contractility in the presence or absence of atropine or TTX was examined. In sham animals, both agonists induced a sustained contraction. Pre-treatment with atropine or TTX significantly reduced only A_2A_R-mediated contractions (−80% and −83%, respectively; Figure 4). In infected rats, CPA-induced contractions were modestly, but significantly reduced at 1 and 6 weeks (−11% and −20%, respectively; Figure 4A) when compared to those obtained following stimulation of A_2A_Rs (−74% and −87%, respectively; Figure 4B). Pre-treatment with atropine or TTX abolished A_2A_R-mediated contractions but not those induced by A_1_R activation at 1 and 6 weeks post-viral intragastric inoculum (Figure 4). Selective A_1_R and A_2A_R subtype-selective antagonists were tested next on excitatory effects induced by a submaximal dose of adenosine (1 mM). In normal ileal tissues, pre-treatment with the A_1_R antagonist DPCPX (0.01 µM) significantly increased adenosine-evoked contractions (+72%, p<0.01; Figure 5A) whereas ZM 241385 (an A_2A_R antagonist) significantly reduced (−45%, p<0.01; Figure 5B) adenosine’s excitatory effects. In contrast, latent HSV-1 infection in the ENS dampened the enhancing effects of DPCPX on adenosine-induced contractions at 1 and 6 weeks (−35%, p<0.01 and +50%, p<0.01, respectively; Figure 5A) post-HSV-1 intragastric inoculation, compared with sham animals expressing adenosine-evoked contractions in the presence of the A_1_R antagonist. Moreover, the antagonizing effects of ZM 241385 further reduced the purinergic excitatory effect 1 and 6 weeks after HSV-1 intragastric administration, compared with sham animals expressing adenosine-evoked contractions in the presence of the A_2A_R antagonist (−25%, p<0.01 and −48%, p<0.01, respectively; Figure 5B).

### Immunofluorescence of Adenosine Receptors, ADA and CD73

**Figure 6:**
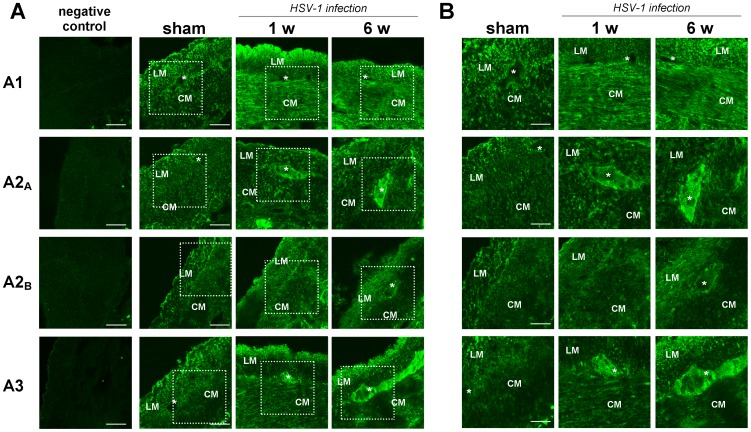
Distribution of adenosine receptors A_1_, A_2A_, A_2B_ and A_3_ receptors in the neuromuscular layer of rat ileum during HSV-1 infection. Representative immunofluorescence staining of adenosine receptors A_1_, A_2A_, A_2B_ and A_3_ in ileal sections from control (sham) and HSV-1-treated rats 1 and 6 weeks (w) after intragastric administration. Immunofluorescence analyses revealed expression of all four adenosine receptors in smooth muscle layers and myenteric ganglia of control ileum as well as changes in their immunoreactivity, both at the ganglionic level and in smooth muscle layers over the time-course of viral infection. The boxed areas (panel A) are enlarged at right (panel B) to show the distribution of the adenosine receptors in the neuromuscular layer. Scale bar: 50 µm (panel A); 25 µm (panel B). CM, circular muscle; LM, longitudinal muscle; *, myenteric ganglia.

**Figure 7:**
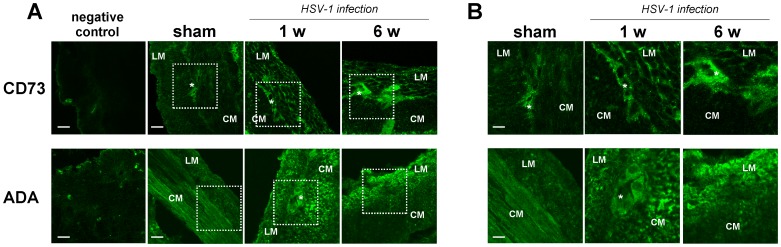
Distribution of the enzymes CD73 and ADA in the neuromuscular layer of rat ileum during HSV-1 infection. Representative immunofluorescence staining of CD73 and ADA in ileal sections from sham and HSV-1-treated rats 1 and 6 weeks (w) after intragastric administration (n = 3 animals for each experimental group). Micrographs showed the presence of CD73 and ADA in the ileal tissue stained with anti-CD73 and anti-ADA antibodies. The boxed areas (panel A) are enlarged at right (panel B) to show the distribution of the CD73 and ADA in the neuromuscular layer. Scale bar: 50 µm (panel A); 25 µm (panel B). CM, circular muscle; LM, longitudinal muscle; *, myenteric ganglia.

**Figure 8:**
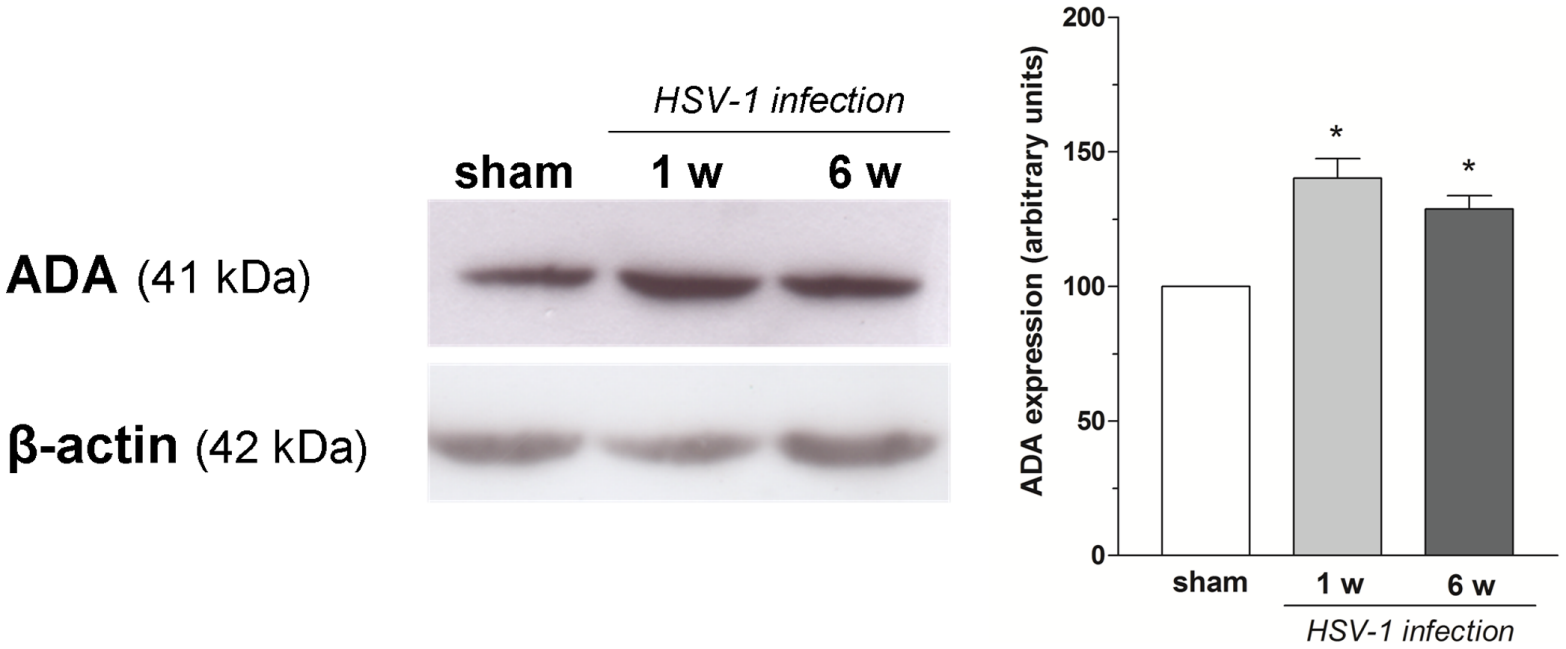
Expression of the enzyme ADA in rat ileum during HSV-1 infection. Representative Western blot and densitometric analysis of ADA expression levels in whole thickness ileum without mucosa from sham and HSV-1-treated rats 1 and 6 weeks (w) after intragastric administration (three tissue samples from n = 3 animals for each experimental group). β-actin was used to normalize loading. *P<0.05 compared to sham animals.

The altered responses prompted us to characterize the expression of adenosine receptors, the adenosine generating enzyme CD73, and the adenosine catabolizing enzyme ADA in rat ileum during HSV-1 latent infection of ENS. Immunoreactivity for A_1_, A_2A_, A_2B_ and A_3_ receptors and for ADA was detected in the neuromuscular layer, whereas CD73 immunoreactivity was barely detectable (Figures 6 and 7). Progression of HSV-1 infection was associated with an altered distribution as evidenced by increased immunoreactivity at longitudinal and circular smooth muscle for A_1_R and A_2B_R, whereas marked immunofluorescence signals for both A_2A_R and A_3_ R were found mainly at the myenteric ganglia level 1 and 6 weeks post-intragastric challenge (Figure 6). Immunoreactivity for CD73 was increased at the level of longitudinal smooth muscle and myenteric ganglia 6 weeks post-intragastric viral administration (Figure 7A). Moreover, ADA immunoreactivity increased within myenteric ganglia at 1 week, followed by the longitudinal smooth muscle layer 6 weeks post-intragastric HSV-1 infection (Figure 7B). Western blot analysis showed a significantly increased ADA expression in the ileum at 1 and 6 weeks after intragastric administration of HSV-1 (Figure 8).

## Discussion

The involvement of adenosine pathways in the pathophysiology of gastrointestinal dysmotility associated with ENS dysfunction, although known, is represented by data which are both limited, and sometimes conflicting [12], [13], [20]. The present study was carried out to determine the effect of a viral infection of the ENS on contractile responses to adenosine, as well as on distribution of adenosine receptors and of its enzymes CD73 and ADA in rat ileum. Our results show that: (i) under normal conditions, exogenous adenosine contracts longitudinal smooth muscle of intact rat ileum; (ii) in the presence of HSV-1 infection in the ENS, adenosine-mediated contractile response by A_1_ and A_2A_ receptors is impaired at 1 and 6 weeks post-infection; (iii) latent HSV-1 infection in neurons influences the distribution of CD73, ADA and adenosine receptors in a distinct manner in ileal smooth muscle layers and myenteric plexus.

Since HSV-1 infection in the ENS determines ileal dysmotility due to impaired cholinergic neurotransmission, we decided to evaluate adenosine’s effect on spontaneous basal contractility and not on pre-contracted preparations. This was done to avoid any influence of the underlying anomalous cholinergic transmission induced during HSV-1 infection of ENS [11]. Exogenous adenosine or related analogues influence cholinergic and tachykininergic transmission, thus impacting on intestinal motility, peristaltic reflex, transit and secretion in the small and large intestines of rodents [12], [13], [20]. In spite of a large body of published studies, the physiological function of adenosine remains to be fully clarified. This is an important point, considering that adenosine-mediated effects have been mainly studied on intestinal preparations pre-contracted with a muscarinic agonist or with electric field stimulation-evoked acetylcholine release rather than on ileal spontaneous basal tone [17], [21]–[24]. Moreover, difficulties in dissecting adenosine-mediated responses arise from concurrent activation of different adenosine receptor subtypes, each leading to opposing (i.e. contraction or relaxation), time- and concentration-dependent responses. Thus, stimulation of high-affinity A_1_ receptors appears to be responsible for purinergic modulation on cholinergic and tachykininergic transmission, whereas A_2A_ receptors may either reduce cholinergic motor responses or facilitate acetylcholine release in rodents [12], [13], [25]. Low-affinity A_2B_ and A_3_ receptors exert inhibitory control by regulating nitric oxide release or by negatively controlling cholinergic activity, respectively [21], [26], [27]. Furthermore, adenosine can produce direct opposite effects also on smooth muscle cells, depending on intestinal tone [28]. In our preparations we observed an increase of ileal basal tone induced by exogenous adenosine. This effect, being partially prevented by atropine or TTX, appears to be mediated by adenosine stimulation via P1 receptors of myenteric muscle cells and of nerve endings, thus influencing excitatory neurotransmission [23], [26], [29]. To investigate the receptor(s) responsible for adenosine excitatory effects in physiological condition, ileal segments were exposed to adenosine in the presence or absence of A_1_R or A_2A_R agonists and antagonists. Differential responses to A_1_R and A_2A_R agonists in the presence of atropine or TTX supports the notion that these two adenosine receptors mediate ileal contraction through different mechanisms: A_1_R activity directly influences smooth muscle contractility whereas A_2A_R modulates cholinergic transmission in myenteric neurons [30]. In particular, nanomolar concentrations of the selective A_1_R antagonist DPCPX, applied to ileal preparations enhanced adenosine-evoked contractions of longitudinal smooth muscle whereas the A_2A_R antagonist ZM241385 reduced the amplitude of adenosine responses. These observations support an opposite regulatory action for the two receptor subtypes in ileal contractile activity under physiological conditions [26], [28], [30].

In our animal model the presence of HSV-1 in the ENS provoked a mild inflammatory reaction, as evidenced by changes in levels of inflammatory cytokines during the course of infection and in absence of any tissue damage. Given adenosine’s important role in the complex crosstalk between enteric neurons and smooth muscle cells and the fine tuning of intestinal motor and inflammatory responses, this model may find utility in undercovering the role of adenosine where motility disturbances of ENS origin are present. The increased amplitude of spontaneous phasic contractions in infected animals confirms our previous observations that HSV-1-induced latent infection of ENS causes an underlying anomalous neuromuscular transmission [11]. However, adenosine was still able to induce reproducible and concentration-dependent contraction of ileal segments from infected rats, with a significant reduction in maximal response. Adenosine itself possesses potent anti-inflammatory properties, and is up-regulated in several models of gastrointestinal disorders such as infection induced by the parasite *Schistosoma mansoni* [12], [13], [17]. The responses obtained by adenosine treatment or by direct activation of A_1_ or A_2A_ receptor subtypes in ileal preparations were affected over the HSV-1 infection time-course. Impaired neuronal acetylcholine release during HSV-1 infection implied a loss of A_2A_R-mediated effects mainly at the level of prejunctional facilitatory receptors. On the other hand, A_1_R-mediated effects were still affected by the presence of HSV-1 in a noticeable, albeit minor way, since they are mainly located on the smooth muscle layer and insensitive to atropine or TTX blockade [17], [22].

The biological actions of adenosine are mediated by four G-protein-coupled receptor subtypes differentially expressed throughout the gastrointestinal tract, and depend on animal species [12], [31]–[34]. We thus asked whether HSV-1 infection affected their distribution in the smooth muscle layers and in myenteric plexus. Following HSV-1 infection of ENS, A_1_ and A_2B_ receptor subtypes undergo a marked up-regulation, particularly at the muscle layer level whereas A_2A_R and A_3_R show a marked increase of immunoreactivity in myenteric ganglia and longitudinal muscle layer. Previous studies have focused on unmasking the role of a single adenosine receptor subtype in pathological conditions. As far as we know, the effect of an intestinal insult on the distribution of all four receptor subtypes has not been considered until now. Our observations are in line with past reports describing a reduced inhibitory control of A_1_R associated with enhanced expression of A_2A_R in the neuromuscular compartment, and of A_3_R in the myenteric plexus of rats with DNBS-induced colitis [21], [22], [35]. Increased expression of A_2B_R has been reported in inflamed colonic epithelium from humans and mice but not in smooth muscle layers, as we found in our ileal preparation from infected rats [36]. Collectively, our findings support the view that, either during inflammation or infection, the distribution of P1 receptors is affected in both myenteric ganglia and muscular layers, confirming their active role in the pathophysiology of GINMD.

Recent evidence indicates that the ecto-enzymes CD73 and ADA represent synergistic checkpoints for regulating adenosine availability to preserve tissue integrity during insult [12], [14], [37]. Is their distribution affected during HSV-1 latent infection in ENS? We and others [14], [38] have shown CD73 expression in the myenteric plexus and smooth muscle cells of normal ileum, where this ecto-5′nucleotidase mediates the formation of adenosine from AMP. On the other hand, ADA is predominantly localized in the mucosal layer [39]. CD73 immunoreactivity was localized to smooth muscle layers of normal rat ileum, becoming elevated both in myenteric plexus and longitudinal smooth muscle layer at week 6 following viral intragastric inoculum. This increase likely supports CD73 action in regulating endogenous adenosine levels, thereby allowing the latter’s receptors to exert anti-inflammatory actions or, eventually, facilitate viral evasion during chronic infection [40]. In line with these findings, we found also an enhanced expression of ADA initially restricted at myenteric plexus during the acute phase of infection (at 1 week) but as the infection becomes chronic (at 6 weeks) this enzyme extends toward the longitudinal smooth muscle layer. An increase of extracellular adenosine levels during adverse conditions has been shown to be associated with higher ADA expression, presumably to avoid possible deleterious effects of long-term adenosine exposure [39]–[41]. Conceivably, in the presence of inflammation or other harmful stimuli adenosine levels could be fine-tuned to create discrete microenvironments, termed “purinomes”, within the enteric neuromuscular layer by means of nucleoside production/inactivation involving CD73 and ADA, respectively [21], [30], [42]. This would ensure the correct functioning of specific receptor subtypes under different pathophysiological conditions [43], [44].

In conclusion, the findings described here demonstrate that adenosine acts as an excitatory modulator of the contractility of rat ileal longitudinal smooth muscle by stimulating P1 purinoceptors on myenteric neurons and ileal smooth muscle cells. Latent HSV-1 infection in ENS affects adenosine-mediated modulation of the complex crosstalk between enteric neurons and smooth muscle cells, as evidenced by the molecular and functional rearrangement of adenosine receptors and related metabolic enzymes. These observations, together with a growing body of knowledge linking the adenosinergic system to modulation of immune/inflammatory processes can provide a foundation for detecting and understanding digestive motor dysfunctions. Ultimately, this will lead to the development of novel pharmacological tools for the diagnosis and therapeutic management of enteric dysmotility.
